# Data science in the design of public policies: dispelling the obscurity in matching policy demand and data offer

**DOI:** 10.1016/j.heliyon.2020.e04300

**Published:** 2020-06-27

**Authors:** Michela Arnaboldi, Giovanni Azzone

**Affiliations:** Department of Management, Economics and Industrial Engineering, Politecnico di Milano, Italy

**Keywords:** Data science, Policy, Big data, Framing, Knowledge management, Information systems management, Information management, Human resource management, Business management, Strategic management, Risk management, Information science, Business

## Abstract

Data Science (DS) is expected to deliver value for public governance. In a number of studies, strong claims have been made about the potential of big data and data analytics and there are now several cases showing their application in areas such as service delivery and organizational administration. The role of DS in policy-making has, on the contrary, still been explored only marginally, but it is clear that there is the need for greater investigation because of its greater complexity and its distinctive inter-organizational boundaries. In this paper, we have investigated how DS can contribute to the policy definition process, endorsing a socio-technical perspective. This exploration has addressed the technical elements of DS - data and processes - as well as the social aspects surrounding the actors’ interaction within the definition process. Three action research cases are presented in the paper, lifting the veil of obscurity from how DS can support policy-making in practice. The findings highlight the importance of a new role, here defined as that of a translator, who can provide clarity and understanding of policy needs, assess whether data-driven results fit the legislative setting to be addressed, and become the junction point between data scientists and policy-makers. The three cases and their different achievements make it possible to draw attention to the enabling and inhibiting factors in the application of DS.

## 1 Introduction

The era in which we live, this “information age”, is brazenly producing enormous volumes of data [1, 2]. Helped by the many innovative technologies and technical tools in play - consisting of a wide range of mathematical techniques, data analysis techniques, visualization techniques, cloud computing and fuzzy sets and systems [3] - these data can be elaborated to build exciting datasets that can be accessed by all. These progressively large datasets contain highly detailed information obtained ever more promptly from different sources, combining data of a traditional, transaction-based origin with those collected either automatically, like the signals emanating from mobile phones and web connections, or on a voluntary basis, like the material we publish on social media [4].

Data Science - the umbrella name given to the innovative use of “analytics” to extract information and insights from these many and diverse datasets [5, 6] - was initially developed for business purposes. It can, however, also be used to support decision-making in the public sector [7, 8], helping us to gain a deeper and more transparent understanding of our world [9], while improving the way we identify and assemble the choices made by people when faced with a number of possible options [10]. Availability and use of analytical information can help, in particular to achieve:

• **greater efficiency and effectiveness** in planning and implementing public policies, as it becomes increasingly easier to understand what each user's expectations are and so “target” any initiative [11, 12, 13].
• **greater promptness** in understanding how phenomena underway in society can require special interventions, for example, by measuring population sentiment - either locally [14] or nationally [15] - or taking field observations of how a given pathology is spreading (through DDD, digital disease detection, Vayena et al. [16]).

Although the potential applications of Data Science (DS) in many fields and sectors have been propounded in a number of studies, in practice these applications are rarely found in government processes or they are, at most, only at the experimentation stage [17]. Examples include the criminal sector [10, 18, 19, 20], taxes and fiscal areas [21, 22] and healthcare services [23, 24].

This paper focuses instead on the role that DS plays in designing public policies seen as “a set of actions that affect the solution to a privacy problem or dissatisfaction concerning a certain demand or opportunity for public intervention” [25]. More specifically, the objective of this article is to investigate how analytics and data scientists can support the process of defining a given public policy, removing the veil of the obscurity from the underpinning process. Three cases are here presented, where DS has been utilized to support the design of public policies. By exploring these cases through action research and endorsing a socio-technical perspective [21], evidence is provided concerning:

• How the process is carried out and what role the various phases of the DS cycle play in the policy definition process, from the initial definition of the problem to the resulting legislative measure;
• What contribution is made by the various actors taking part in policy definition processes, including the DS team, project managers and politicians;
• Finally, what is the contribution of DS to policy definition processes, and which factors influenced the various outcomes. Here the contribution is measured by examining whether - and how effectively - elaborated data can be used to put in place a public policy (in the form of legislative measures and/or structural investment and/or intervention to public services). The effects of the policy on the quality and quantity of services delivered have not been examined, in part because of the overly lengthy time necessary for any impact to be felt.

To set out our argument, the paper is articulated as follows: Section 2 contains the review of previous studies and an explanation of our conceptual perspective; Section 3 lays out the methodology, the findings are then presented and are followed by the discussion and conclusions.

## 2 Data science and public policy: dispelling the veil of obscurity

Data Science (DS) is an interdisciplinary field concerned with extracting value from bodies of heterogeneous data, moving from the process of acquiring data to that of data analysis and visualization [5]. As noted by Rempel et al. [6], DS encompasses “both the technical practices of data usage and data technology development, but also the ways in which data science interacts with and informs social and political practice” (p. 570). DS is based upon two pillars. The first pillar consists of data where particular emphasis is given to big data, although DS also has the potential of generating new insights from data sets that do not always encapsulate the 3Vs of Volume, Variety and Velocity^1^ [26]. The second pillar is the elaboration process, consisting of the cycle of phases required to transit from raw data and generate knowledge. Public sector interest in DS is very high, and a number of studies are now making claims about its importance and potential and, through experimentation, they provide empirical evidence about its potential value [10, 17, 27]. Most of these tests, however, do not bring in the decision- and policy-makers, leaving open the question of whether the resulting information is useful or not for making public decisions. In a small number of studies, the DS process has been explicitly investigated from a socio-technical perspective, paying attention to both the technical aspects and the role of the actors [6, 10, 12, 19, 20, 21, 23, 28, 29, 30, 31].

These studies reveal the importance of how data scientists^2^ interact with data that can ultimately be used to generate knowledge. Despite this, the empirical studies currently available do not focus on the policy-making processes, an area where the technical aspects and the dynamics of the actors involved are both more complex. In their position paper, Janssen and Helbig [32] highlight this socio-technical complexity and the impact of advances in DS, and they specifically draw attention to the new roles and capabilities required for policy-makers. They point out that: “In essence, policy-makers have to have knowledge about the (im)possibilities and limitations of computational instruments and methods, whether a policy model is valid, how to use big and open data, know how to integrate instruments and methods in public discourse and understand the wishes, needs and behaviors of the broad range of stakeholders” [32].

In our study, with this auspicious thought in mind, we have investigated how DS is used to define policies in real practice, opening the black box of the data, processes and roles involved. The paper endorses a socio-technical perspective, giving importance to the technical aspects of DS, in the full knowledge that the generation of knowledge is a socially constructed process [33] in which the contextual and relational aspects are of pivotal importance [12, 32]. The theoretical background that frames our study can be traced to previous research that points out the problems of relating the “offer” provided by data scientists, in terms of data and models, with the “demand” from the policy-makers. DS is supposed to generate new knowledge, but the generation process does not take place in a bubble of objectivity [33] but is a path where the actors’ subjectivity and their relational engagement matters. In the age of big data and analytics, this process becomes even more complex, because of the broader range and diversity of the competences and phases needed [34, 35, 36, 37, 38].

At the technical level there are two main problems. The first is linked to the phases, and specifically to the higher complexity of the process, whereby data scientists make the transition from often large volumes of miscellaneous data to synthetic indicators, frequently working their way through a series of arbitrary decisions concerning the elaboration methods or how such data are visualized [30, 32]. A second distinctive technical problem is that of information scattered among several public administrative bodies, when this information could potentially be used to provide solutions to policy problems [17, 39]. One implication of this state of affairs is that the various players must carry out negotiations before the data can be shared, with all the associated “technical” problems, including that of privacy, and the social pressures linked to each stakeholder's desire to retain power and control over the data [4, 10].

This higher technical complexity is compounded by issues associated to the actors and their relational context and background. The first problem is that data scientists and policy-makers lack a “complete” set of competences. As shown in previous studies [12, 32], in today's DS age, policy-makers are expected to own a substantial capital of capabilities that include both soft skills, such as the blend of leadership and organisation known as orchestration, and hard skills, such a solid technical background. At the same time, data scientists have a rather meagre skillset in the sphere of public processes and policies, generally preferring vertical skills and specializing in one of the single phases of gathering, analysing and reporting on the data [10].

The complexity in data and phases, which is emphasized by misalignment in communication caused by the players' different backgrounds and conceptual frameworks, has given rise to the second relational issue: the potential mistrust in the data to be utilized [40]. When decision-makers are not confident that their data, or the origins of the data, are solid, and are unsure about their impact on public opinion, it is unlikely that they will use the data to build policies. Distrust and over-prudence towards DS is then heightened by the potential negative vibes of public opinion [6], especially in western countries, as a reaction against their governments’ use of detailed analytical information about individual people. For example, LaBrie et al. [41] on comparing the position taken by two groups of students, one Chinese and the other from the USA, pointed out that, for the USA students - differently from the Chinese group - concern about loss of privacy was an “overriding” factor compared to any potential benefit of such analytic information being used by governments to improve the quality of their policies.

The socio-technical issues described above generate a “lack of demand” and “information misfit”, which are also interdependent matters. On the one side, the very fact of improving the quality of decisional processes could conceivably act as a legitimate mandate for governments to use this analytical data, and so contribute, for example, towards overcoming critical public opinion concerned with any insidious loss of privacy [16]. On the other side, the presence of a strong “demand” on the part of decision-makers can encourage collaboration between the various authorities that “own” the data.

Removing the veil of obscurity from the process and being able to identify the factors that can best stimulate the policy-makers’ demand and improve the quality of analytical data underpinning decisions is, therefore, essential for transforming the potential benefits of data science applied to public policies into tangible assets. In this study, we observed two practices renowned for creating obscurity that are often exercised by data scientists. These are filtering and framing [30]. In the first of the two, filtering, data are first selected and, through techniques that elaborate a series of attributes and properties relating to the data, are transformed into information and, subsequently, into knowledge available to the decision-makers. In the latter, framing, data is contextualized and then communicated in a form that can be understood and trusted, ensuring that it will be put to use. Filtering and framing are at the basis of this investigation into how analytics can support (or not) the process of determining public policies, revealing both the enabling and the inhibiting factors.

## 3 Methods

By adopting a qualitative methodology, we could gain greater insight into our problem, that of examining whether analytics can help the process (or not) of determining a public policy. A qualitative method is also expedient for thoroughly examining the associated enabling and undermining factors, an area that, so far, has been largely unexplored. Within this framework, the study was conducted through action research, a process referred to by Rapoport [42] in his seminal work. The distinctive feature of this method is that it “aims at to contribute both to the practical concerns of people in an immediate problematic situation and to the goals of social science by joint collaboration within a mutually acceptable ethical framework” [42]. In this paper, the authors present the results of three cases carried out within the action research model, where the external actors involved welcomed and stimulated the use of DS for determining policies. The three cases are:

• E-mobility, where data is used to set out the process of developing a nation-wide infrastructure for charging electric vehicles;
• Casa Italia, where data is used as part of the process to determine prevention policies to protect housing stock from natural hazards;
• Urbanscope, where data is used to define policies working within the internationalization process in an urban area.

**Figure 1:**
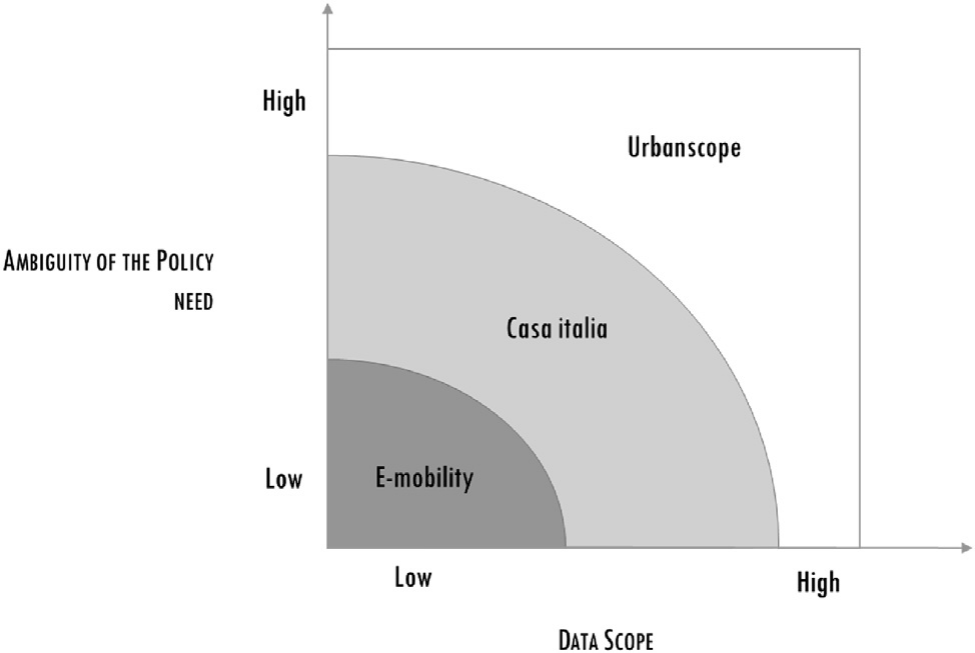
Case heterogeneity.

**Figure 2:**
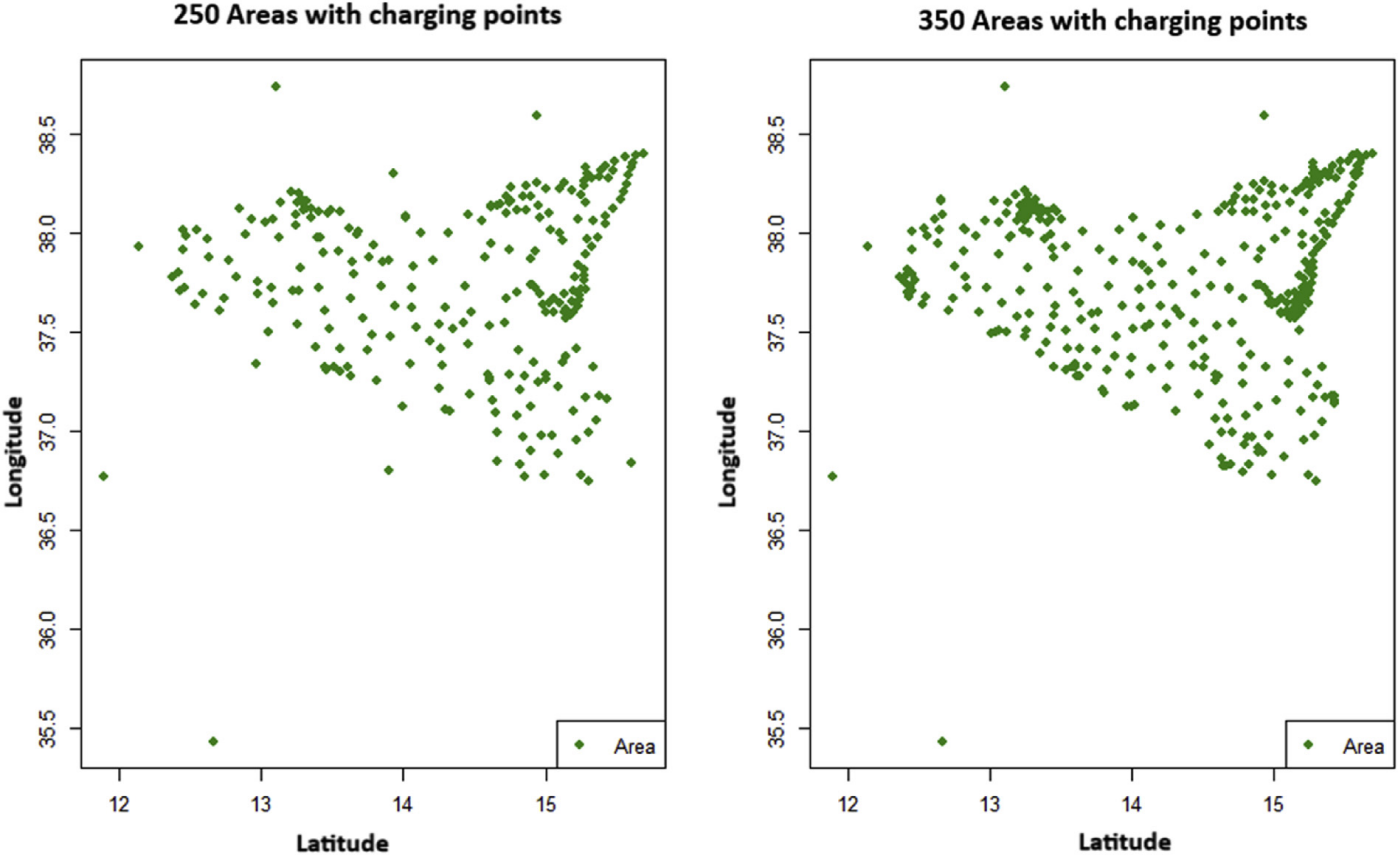
Example of data mapping for E-mobility.

The three cases vary in terms of the scope of the data (with respect to Volume, Variety and Velocity) and the clarity of the policy needs. In Figure 1, the cases are mapped along these two dimensions. E-mobility has the clearest policy need and makes the “simplest” use of data, while the opposite is true for Urbanscope, which fields a vague policy and a set of data with all the characteristics of big data (Figure 2). Casa Italia is a half-way in-between case.

The same research protocol was adopted in all three cases^3^, featuring a cyclical process of five phases consisting of diagnosing, action planning, action taking, evaluating and specifying the learning outcome. This cycle had to be adapted to the timing and scope of the three cases. In terms of the project's duration, E-mobility took the least time, with the process lasting a total of six months, including the initial work to define the problem. Casa Italia took 16 months; the initial diagnosis to examine how to tackle the problem started in September 2016 and the project was concluded by the end of 2017. Urbanscope ran for more than two years; the project began in February 2014 and ended in July 2016.

The authors of this paper were involved in the three cases, taking on specific roles within the projects. in E-mobility, one of the authors coordinated the DS team and the second author acted as an external observer; in both Casa Italia and Urbanscope, one author was the project manager and the other was involved as an expert in public management. In all three cases, the scientific setting and management was “owned” by the authors, meaning that the same protocol could be applied. The phases of diagnosis and action planning were based on multiple sources of evidence, consisting of formal interviews with the relevant actors, archival material, database analysis and participation in public and private meetings. Interviews were the pivotal element of the diagnosis phase; they were carried out face-to-face and lasted 1 h on average. Table 1 gives the list of actors interviewed. A key role in E-mobility was played by a utility company with links to the government, here referred to as Power.

**Table 1:** Informants in the three cases.

Urbanscope	Mayor of Milan Vice Secretary at the Chamber of Commerce of Milan Head of Cultural Activities at the Municipality of Milan Head of Marketing at the Municipality of Milan Manager of the Statistics Office at the Chamber of Commerce of Milan Journalist and member of a citizen's association politically active in Milan Entrepreneur and member of a citizen's association politically active in Milan Academic and member of a citizen's association politically active in Milan
Casa Italia	Prime Minister of Italy Head of Personnel in the Prime Minister's office President of CNR (the National Research Council of Italy) Senior researcher at CNR President and two managers at ENEA (the Italian National Agency for New Technologies, Energy and Sustainable Economic Development) Senior researcher at ENEA President of INGV (the National Institute of Geophysics and Volcanology of Italy) Senior researcher at INGV President of ISTAT (The Italian National Institute of Statistics) Head of territory statistics at ISTAT Senior researcher at ISTAT
E-Mobility	Head of the Technical Secretariat at MIT (Ministry for Infrastructure and Transport) Senior Manager at MIT CEO at Power Head of the Italian Distribution Network at Power Development and Innovation Unit at Power Head of Corporate Affairs at Power Marketing manager at Power Communications manager at Power

The two phases of action taking and learning were based on an iterative process, where the subject matter was debated positively within the DS team and with external actors. The rigour of this interaction was maintained by consistently preparing in advance a systematic report of the proposals and matters to be discussed, allowing the research team to keep track of the reactions of the various parties involved and of where revision was needed. Further details about the DS team and the involvement of policy-makers are attached to the case results.

The data collected were catalogued and archived according to the three dimensions to be investigated: the Processes, Roles and Critical Factors linked to matching the DS demand and offer. With respect to the processes, the researchers took as reference the phases of big data cycle (ref omitted): data acquisition, data analysis, data integration and fusion, modelling and visualization. We associated the relevant material from different sources to each phase, first analysing the evidence for each case and then employing a cross-case perspective. Tables highlighting the technical elements linked to data, techniques and human interaction (not shown) were introduced to facilitate the second order analysis. Our second dimension, roles, was analysed mainly through human interaction. This exploration was carried out through abduction: starting from the roles that had emerged in previous studies [12, 32], we added the emergent roles by cross-checking the cases. In this phase, data were analysed using a within-case matrix and a number of cross-case matrices. The process phases were set out on the vertical axis of the within-case matrix (Table 2), which was then used to map the other two dimensions (roles and critical factors). The cross-case matrices were, instead, used to compare each column for the three cases in order to highlight the differences and similarities (Table 3).

**Table 2:** Within-case matrix (example from the Urbanscope case).

Phases	Roles	Critical Factor
Data acquisition	Need for a negotiator to acquire data in a sustainable and secure way.	Need to ensure continuity in data collection
Data analysis	High need for a person to take on the role of interacting with the policy-maker and gain a better understanding of the data that are relevant	Lack of clarity concerning the policy requirement
Data integration and fusion	Need for a person with the role of capturing relationships and the potential of data in decision making	Scattered data which had never been collated, meaning that the policy-makers were unprepared to cope with complex analyses
Modelling and visualization	Need for people with the role of selecting the best communication tool to stimulate the policy-makers’ ideas	Compress multiple dimensions in a simple way

**Table 3:** Cross-case matrix (an example for data acquisition).

Phases	Roles		
	Urbanscope	Casa Italia	E-Mobility
Data acquisition	Negotiator for economic sustainability and security	Negotiator for inter-organizational collaboration in the public sector	Negotiator for inter-organizational collaboration

With regards to big data, large sets of data were used in each case, as described in greater detail in the results section. The Urbanscope case is presented by way of example, as this case has the most variety, with the focus here being on Twitter data. Twitter's API (Application Programming Interface) platform was used to download the tweet-related data that was required for the analysis, consisting of the payload for each tweet, as well as the body of the tweet message and a series of useful metadata, ranging from language and timing to links, tags and so forth. Geographical metadata giving the exact latitude and longitude of the location from which the tweets were sent, if available, were used to assign the tweets to their appropriate district of Milan. The language in which the tweets were written is detected automatically by Twitter, and when the algorithm determined that more than one language was used, the tweet was recorded as written in an “undefined language”, and these tweets were not included in the dataset. Apart from removing tweets using abusive or vulgar language, there was no further filtering or cleaning before proceeding with the analysis, and the tweets removed were still counted for statistical purposes. In all the cases, the mathematical analysis was carried out using the R programming language and, in the Urbanscope case, for example, the R “igraph” package was used to analyse mobile phone data.

The third dimension, critical factors, was covered by preparing a synthesis of the problems in matching the demand and offer. The processes, data, roles and critical factors were then re-analysed for external validity [43], allowing the three propositions to be defined, as reported in the discussion. For this last step, the research team employed about a dozen cross-case matrixes (see Table 3 for an example), comparing the three variables.

## 4 Results

This section analyses the three cases, each divided in three sub-sections according to the type of data, the roles coming into play and the critical factors that emerged when matching the demand and offer of data.

### 4.1 E-mobility

The first case was set up at the behest of the Ministry of Infrastructure and Transport (MIT) to tackle the scarcity of electric vehicles in Italy, where the numbers circulating are much lower than in many other European countries. One of the reasons for this is the limited distribution of charging stations across the country. Until 2016, Italy's policy on this matter was reduced to a series of official announcements offering incentives to local town and city authorities if they installed charging points. The result of all this was a smattering of charging stations distributed in a haphazard fashion. The lack of infrastructure - whereby drivers could travel the length and breadth of the country without worrying about if and when they could recharge their cars - was a break on the spread of electric vehicles. According to the available data, the cost of installing the necessary infrastructure was estimated at 1 billion euros. Within Italy's economic context, this level of investment could not easily be borne by the public purse.

#### 4.1.1 Roles

The problem was discussed with a utility company with links to the government, which is referred to here as Power. What emerged from discussions between the Ministry for Infrastructure and Transport (MIT) and Power, was that Power could take on an active role in developing a network of charging stations and help MIT to identify the layout of the network and possibly take part in its implementation. Power, as a company producing and distributing electricity, had a clear interest in powering up a national infrastructural network. Indeed, it was most probably Italy's prime stakeholder because, differently from other European countries, the leading “national” car manufacturer (the Fiat-Chrysler group FCA) had yet to develop a proactive policy for electric vehicles. In addition, by being under public control through the state shareholding, any proposal set out by Power would automatically be considered “more reliable” in the eyes of the public authorities.

Power decided to tackle the problem by nominating an external DS group, asking it to build models that could determine “the charging infrastructure necessary to reach any place in Italy from any other place in Italy in an electric vehicle”. Power retained overall project management through its Development and Innovation unit. The project manager acted as interface between the DS team and the policy-maker (MIT), ensuring that the outcome would address MIT's needs and also that the team would be able to deliver this outcome within four months, the time agreed with MIT.

The workgroup consisted of data scientists, experts in the specific area (electrical engineers), experts in system engineering (to help with the instructions, construction and general blueprint of the system). In total, three senior researchers and four junior researchers worked on the project.

#### 4.1.2 Data

Time restrictions meant that the researchers had to rely on public sources of data from the beginning. The sources available were mainly the following:

• The breakdown of the roadway system in Italy, using geo-referenced data;
• Traffic flow on the motorway system (number of vehicles circulating on each stretch of motorway, at different times of the day and different periods of the year), placed in the public domain by motorway operating companies;
• Daily city traffic for business purposes, using official Italian census data. From the data, it was possible to establish how many people travelled for work between any two towns and/or cities every day in a private car.

There was no information on local traffic flows for reasons other than business, or on urban and extra-urban traffic. With regards to public sources, the phase of data acquisition was the simplest, as it was not necessary to enter into negotiation or make any agreements about transferring data.

#### 4.1.3 Critical factors in matching demand and offer

Although the policy at stake was clear (design an electric charging point infrastructure to encourage the use of electric vehicles), modelling the problem was not so simple and it brought into play a number of factors that straddled the DS, policy and public administrative body arenas. Using the available data, the question could be addressed according to different scales and/or constraints. With regards to scale, designing the infrastructure network can be seen as a two-level exercise: (1) at “macro” level, to determine, for example, the number of charging stations in each town or city or in each region; (2) at “micro” level, to decide, for example, exactly where to locate each charging point (its precise address). With regards to the constraints, these boiled down to the acceptable time it takes to charge an electric vehicle: (1) would it be enough to have access to a charging point and so avoid being left stranded or (2) should the charging times be similar to the time currently necessary to re-fuel a car with an internal combustion engine?

The working procedure chosen was an iterative-type process. The starting point was to elaborate very detailed data analysis models and search for the minimum number of charging points necessary and their precise location. The figure below shows how these would look for Sicily.

Going deeper into the analysis, it became apparent that this solution was not acceptable from an administrative angle. For example, the location of the charging points cannot be decided by government, but must be resolved locally by each town or city authority. The data, therefore, could not fully “justify” the hypotheses put forward.

Through their continuous interaction, the workgroup and Power were finally able to elaborate a model of the problem, in a form that was compatible with the data and coherent with the requirements set by MIT. These were to identify the number of charging stations to be installed in each Italian municipality and along every stretch of the motorway system, allowing drivers to go from any one point of Italy to any other without having to take more than 15 min to charge their vehicles. For extra-urban roads, without precise information, the objective was simply to install a charging station every 80 km, for purely “emergency” purposes.

In reality, modelling the problem led to a slight change to Power's initial idea, which had been to prepare a detailed design of the infrastructure with the exact location of each charging station. The solution proposed was, however, in line with the policy needs and compatible with the times set, together with the legal and administrative constraints relating to its subsequent implementation. Power's revised proposal was hence accepted by MIT. By re-framing the problem, it was possible to identify the necessary investment for building a network that was coherent with the objectives. This cost came out at much less than previous estimates (about 200 million euros in total), meaning that the project to develop a nation-wide network of charging stations could get underway. The work to implement a network along these lines began in late 2017.

### 4.2 Casa Italia

The second case, Casa Italia (“the House of Italy”) was the direct enactment of a commitment taken in 2016 by the then Italian Prime Minister, Matteo Renzi.

The government's objective was to define a multi-year plan of projects to radically address the problem of natural hazards, and, within the larger frame, identify a series of priorities to tackle immediately. The problem is certainly more complex because of Italy's particular geographic features, with its many sources of natural hazards and the dangers linked to earthquakes, volcanoes, flooding and landslides. Alongside these are the many accumulating effects that are pitched into the limelight by events such as the recent natural disaster of Rigopiano, where the combination of earthquake and landslides destroyed a hotel causing various casualties.

#### 4.2.1 Roles

The Prime Minister played a pivotal role in the initial stages, defining Casa Italia as an innovative undertaking and appointing one of the paper's authors as project manager. In this role, the author had the freedom to set out the research operations and select the most appropriate resources. In line with Mr Renzi's intentions, the project manager brought in a team of scientists with expertise in various fields. These domain experts included natural risks experts, urban planners, public management experts and architects. In addition, since the plan was to introduce a policy underpinned by data, statisticians and quantitative economists also came on board. A total of 17 researchers were committed to the project.

While the project was running, a major political event took place. In the aftermath of the rejection of a referendum promoted directly by his government, Mr. Renzi resigned and a new government took office. After a two-month freeze, the Casa Italia project was given the go-ahead. by Italy's next Prime Minister, Paolo Gentiloni. Mr Gentiloni stated publicly that he considered the prevention policy to be a cornerstone of the safety and the economy of Italy.

#### 4.2.2 Data

While there were copious amounts of data available on natural hazards, they came from a wide spectrum of sources, so the topic fully mirrored the complexity of what are known as big data. The decision was taken very early on to reduce the complexity of the problem and make sure that the quality of the information used as input was good. The focus was on the following:

• the database had to be under the direct care of an official, nation-wide research centre;
• the database had to cover the entire territory of Italy;
• the spatial resolution of the database had to be such that specific local features could be identified and compared.

Even with this work, however, the researchers came up against a “silo”-type system where information relating to the different elements of a danger or hazard were in the hands of different public authorities. Data on hydro-ecological hazards were kept by the Ministry for the Environment, those on building vulnerability by ISTAT, the Italian National Institute of Statistics and those on seismic threats by INGV, the Italian National Institute of Geophysics and Volcanology. The aggregation units for the data sources varied (water catchment areas for hydro-ecological data, municipalities for buildings and so on) and it was, therefore, not possible to take the open source information produced as a matter of course by all public sector bodies and use it automatically. Rather, the researchers had to go back to the basic data or ask the public bodies to elaborate the data specifically.

#### 4.2.3 Critical factors in matching demand and offer

A first critical factor, linked to source selection, was the fact that the framing and filtering revolved around a major decision, that of what the best unit of analysis should be. Many units were considered, starting from single buildings up to the level of province (administratively, a province is the intermediate layer between region and municipality) or even all twenty Italian regions. To find the best option, the team carried out a simulation, asking the central agency for a sample of data not usually in the public domain, meaning that simulations and debates with policy-makers were backed by real data. The project manager was key here in explaining the policy needs and constraints to the DS team and so reduce data redundancy. After several simulations, the team, with the agreement of the policy-makers, decided to opt for the municipality level. This unit of analysis has two main advantages:

• It allows data originally built for different purposes and end uses to be merged;
• It is easily recognized as a local administrative unit, so that users - the general public, administrators, policy-makers - find it easier to make comparisons between aggregated and integrated data that refer back to the statistical unit.

A second critical factor emerged when the team moved from experimenting on a partial dataset to constructing an entire nation-wide database by fusing data. On entering in this phase, the central agency expressed some reservations, mainly about privacy issues. The open data made public did not have the level of detail necessary, hiding key aspects essential to the data fusion process, so a different dataset was made available on an exceptional basis. This critical factor was handled by the project manager, with the government's formal commitment.

In this phase, the project manager's role was particularly important, having been invested with trust in this matter, including publicly by the government (which had clearly indicated that the programmes were strategic for the entire nation). Two positive factors in particular came into play:

• The institutional acumen of public sector authorities, to whom it was obvious that the request came with the backing of the Prime Minister;
• In an even greater measure, a feeling among public officials that their work of processing data had a purpose, since in the past, data were produced without knowing if such data were wanted.

The project manager, however, realized that he had neither the time nor the expertise to maintain constant interaction with the data agencies, and he therefore asked a member of the DS group to take charge of the data acquisition issues. The expert appointed was a statistician who, early on in the study, had demonstrated excellent interpersonal skills, these being considered crucial for interacting with other institutions.

A third critical area was the difficulty of transiting from the big data processed to synthetic indicators and information that were consistent with the identified problem and the legislative measures to be subsequently implemented. Working with such huge volumes of data meant that the processes of filtering and framing became highly interactive. This surfeit of data encouraged the data scientists to conduct a wide number of analyses and elaborate many indicators, without determining a priori whether such processing was coherent with the policy demands. A “trial and error” process was used to go from an “over-surplus of data” to a result that could be translated into a policy. Going through the data scientists’ work with the help of visualization techniques, the project manager was keen to find any element in the data produced that was in line with the policy issues, leading him to ask the data scientists to conduct further analyses. The process ended when they came up with a criterion to select buildings that the policy-makers could understand and justify, and, at the same time, one that could be defined clearly enough to be inserted within a legislative measure.

**Figure 3:**
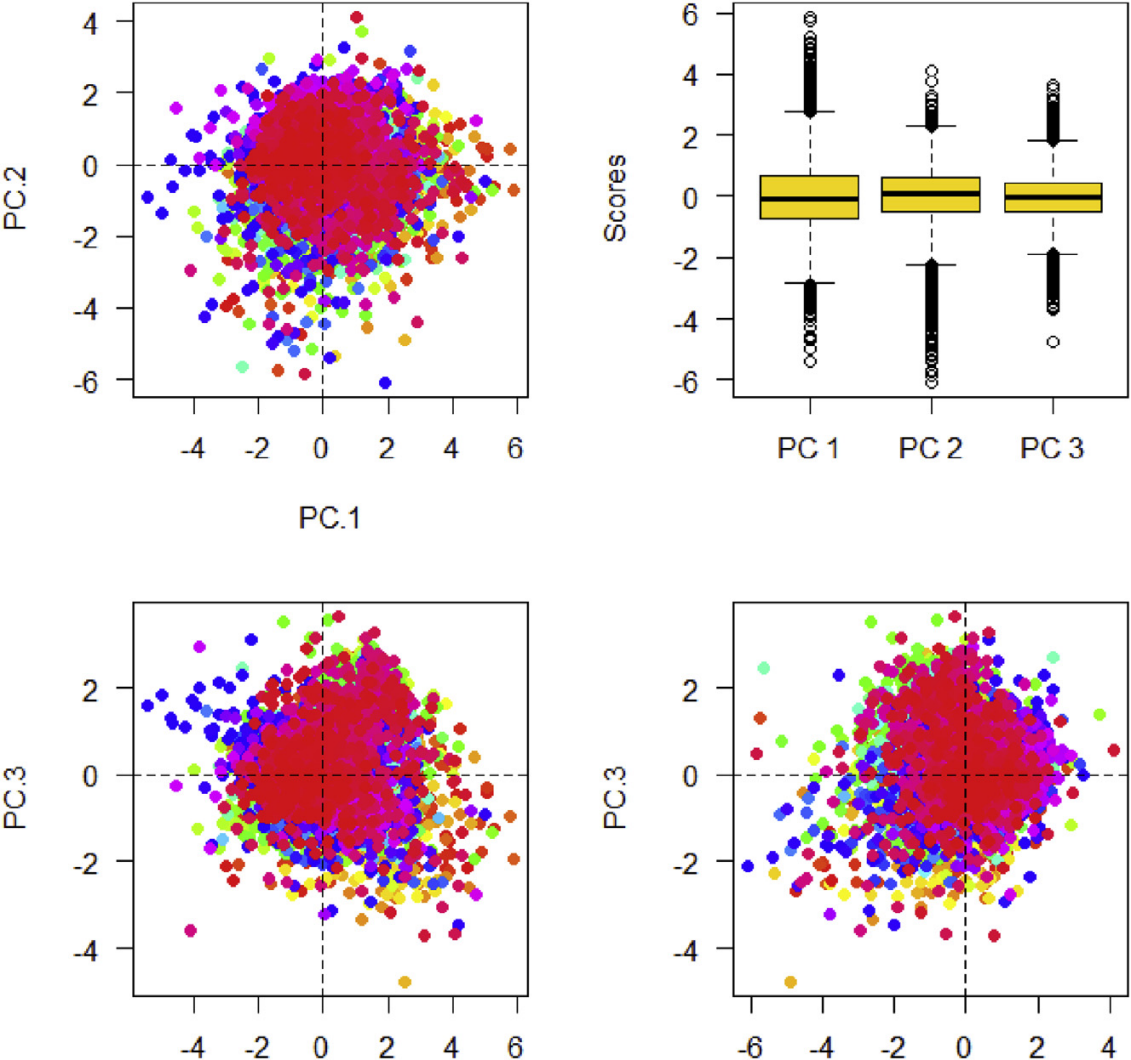
Initial representation of building vulnerability.

An example of this is how buildings that needed work on them most urgently were identified (within 24 million house maintenance interventions in Italy). The data scientists’ preliminary analysis, based upon information about the existing buildings, led to creating maps such as those in the figures below, which are difficult to present to a policy-maker and even the project manager did not find them easy to read (see Figure 3).

**Figure 4:**
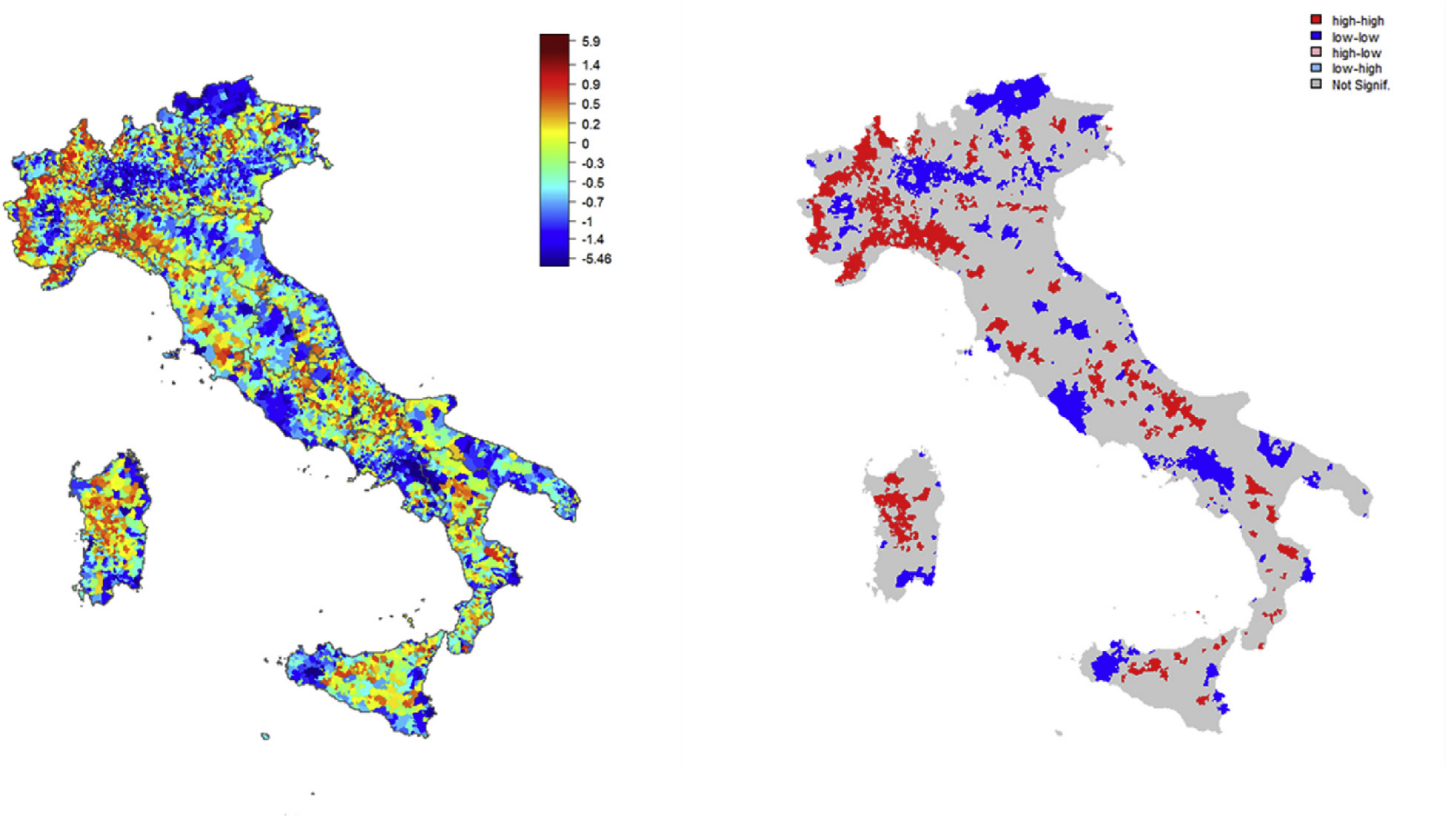
Graphic representation with geo-referencing.

By discussing the matter further, these maps were then “translated” into something more intelligible (see Figure 4).

The last step consisted of “translating” everything into the right language for a legislative measure, using the appropriate criteria and words, as shown below for buildings at risk:

“loadbearing masonry constructions, built before 1970, in municipalities with a seismic acceleration factor >0.25”.

In May 2017, the first legislative measure on the subject was approved, where the state would cover the full costs of diagnosing the seismic vulnerability in buildings with these features (with required public funding of just over 200 million euros).

### 4.3 Urbanscope

The third case is a good example of a self-organized process [17], where the general public collects data to stimulate policy-makers into acknowledging and addressing a need, in this case, that of the internationalization processes taking place within a given urban area. The idea came from a voluntary association of private individuals in Milan, bringing together entrepreneurs, professionals, managers, journalists and, more broadly, the city's citizens, in their commitment towards improving life in Milan and the city's international role.

#### 4.3.1 Roles

The main role in this project was taken by an association member, who acted as the intermediary^4^. Policy-makers, while identified as Milan's politicians, were not involved formally at the beginning, although several association members were connected to the local government of Milan and with whom the project was shared.

With a desire to gain new insights and ask new questions, the association enlisted experts from different fields to ensure full DS coverage in computer science, statistics, design and public management. A total of eight people worked on the project and the senior expert in public management acted as project manager.

The policy-makers were officially brought in after the team had been working on the project for a year, when the group was ready with a web-based dashboard to present the information generated. The policy-maker in question was the mayor of Milan, who was shown the results of the work and the methodology adopted.

#### 4.3.2 Data

The data used to build evidence had all the typical features of Big Data, i.e. Variety, Volume and Velocity. The screened data came from three types of sources. The first consisted of social media data, in this specific case, Twitter, Foursquare and Instagram. For all three, the group used public APIs (Application Programming Interface) to collect user posts via two search methods, these being posts geo-localized in the urban area of Milan and posts where there was a reference to “Milan”.

The second main source consisted of the official census and administrative data about Milan and other similar cities in Italy and Europe. While it was easier to collect these data, they were not particularly useful for gaining new insights, given their level of aggregation, which differed from country to country. The third source consisted of anonymized mobile phone data, which were provided through a private agreement with a telecommunications company. These data were incredibly interesting for their completeness and velocity, however, the agreement stipulated that the data could not be made public or accessed on a continuous basis, for reasons of privacy.

#### 4.3.3 Critical factors in matching demand and offer

The urban policy case raised two main critical factors, linked to the two phases of data acquisition and data visualization. The main problem for the project was to acquire data from both open sources (specifically, social media data) and private sources (specifically, mobile phone data).

Social media were the richest source of information and so very promising, being apparently open, but instead they gave rise to several problems. While public APIs were perfect for experimentation purposes and even for making internal organizational decisions, several critical issues emerged when moving to the field of public policy. The first was continuity in data collection, necessary for observing changes concerning the beneficiaries of a policy. The second was the statistical significance of social media data in terms of their solidity for backing a choice in policy. Public APIs are not totally transparent about the coverage of data extracted and this situation creates problems in valuing their completeness for a specific target of beneficiaries. Regarding private data, in particular mobile phone data, the data provider was again reluctant to supply data on a continuous basis due to privacy reasons, as it would have been too risky for them in terms of legal compliance.

After realising that there were these two problems, the project manager asked two team members (a computer scientist and a management expert) to screen several different solutions to the situation, but without setting up a more formal responsibility over the data acquisition process. Although several paths were pursued, including that of paying for data, after months of negotiation no agreement was reached, primarily because of the level of detail requested by team, which the providers considered too high. Indeed, the providers professed privacy reasons for their stance, as well as those relating to business: “we do not sell data, we sell analysis and consultancy” (in the data owner's words). At this stage, the association asked the project manager to go ahead with the data available, because they did not want to extend the research time and delay presenting the results to the policy-makers.

**Figure 5:**
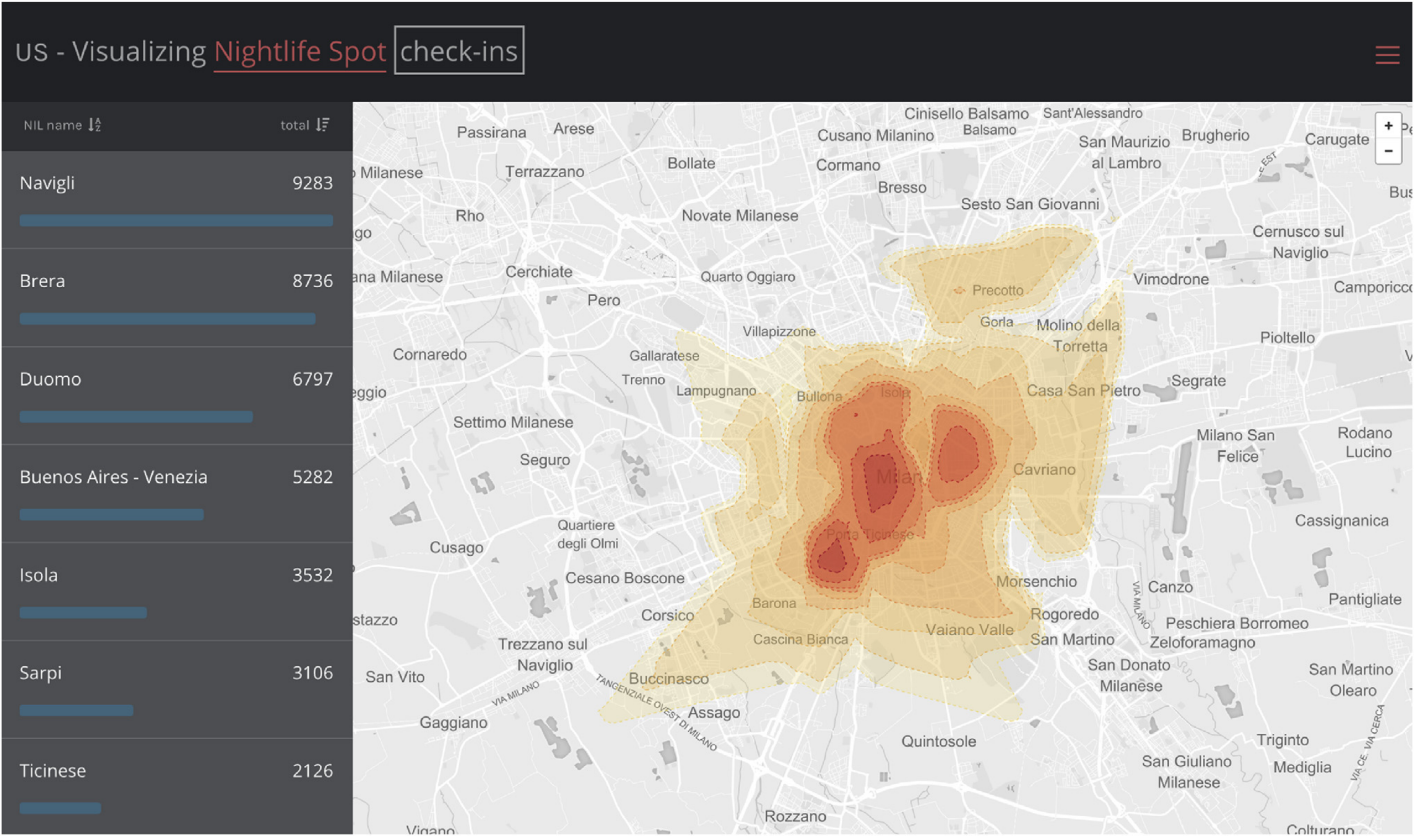
Heat map of nightlife venues drawing the greatest turnout.

The second critical factor was linked to the visualization of data. Urbanscope data involves the highest complexity in terms of Volume, Variety and Velocity. Presenting these data was never going to be easy and, although full of new information about the city, the first display shown to the association, were not, apparently, “inspiring”. The data scientists accordingly embarked upon a long process of interaction, where the association acted as translator to fully explain the policy need to the DS team, and the project manager came in whenever there was any uncertainty or ambiguity during the data analysis. The DS team proposed a refined visualization output, which were the result of filtering the data and framing them in a way that could communication the information clearly. As in the previous cases, the most effective visualizations include geo-referenced data, as shown in Figure 5.

**Figure 6:**
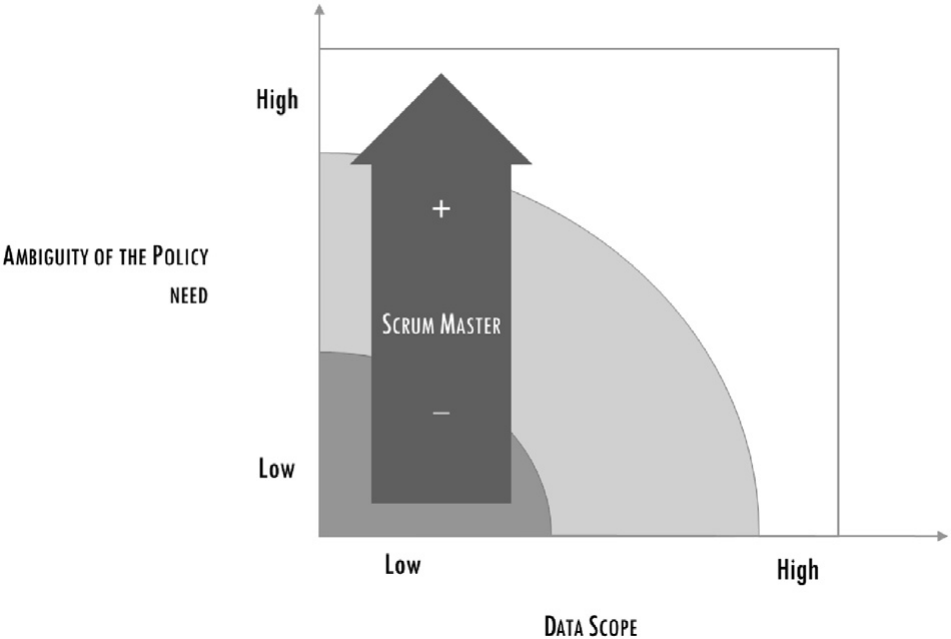
The increasing importance of the Scrum master.

**Figure 7:**
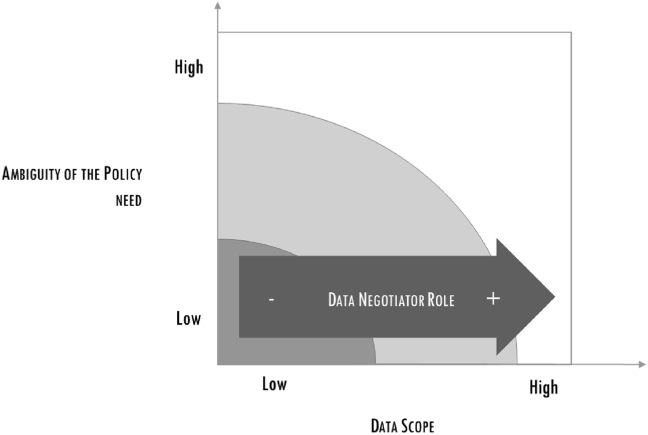
The increasing importance of the data negotiator.

**Figure 8:**
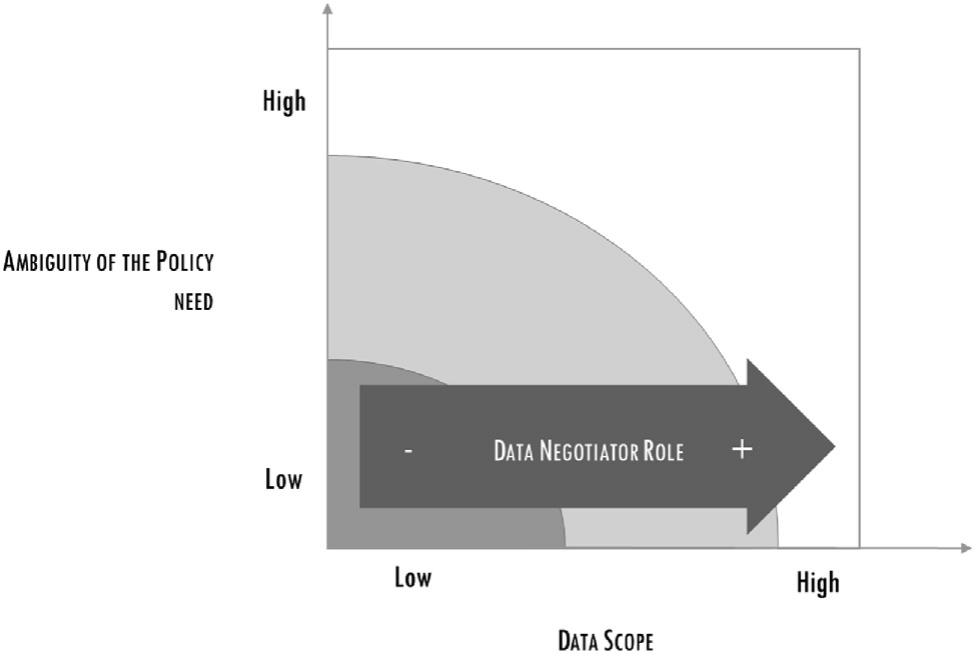
The increasing importance of the visualization phase.

These problems created tensions among the DS team, exacerbated by the mounting pressure of the project manager: this was seen by the team as a danger for the quality of the output, as it reduced the time necessary to framing and filtering data. At this stage the project manager asked a member of the team (with a management background) to take charge of the DS group facilitation. This enrolment favoured a smoother path towards the final delivery: the project manager remained accountable for the final output and for the interaction with the association; the “facilitator” interacted with the project manager, filtering information for the DS team. The last part of the project was very intense, for the desire to find the best framing and the optimal filtering of data and analysis. However, the decoupling of the role of the project manager, with the introduction of the facilitator helped reducing tensions and respecting time and quality requirements (see Figures 6, 7, 8).

The results were shown to the policy-makers; these were first sent privately and then were presented at a restricted public meeting. The policy-makers reacted very positively and all the effort put into the visualization work was rewarded: the policy-makers could understand the data perfectly and were able to ask for some alterations to be made. The use of data for policy-making purposes was, instead, not completely successful given that the work stops at the association level, and up to now the data have not been used by the policy-makers. On several occasions, Milan's policy-makers have expressed a generic interest in using the data and web application, but so far, they were unable to manage the complexity of the system, preventing any further practical policy usage.

## 5 Discussion

The three cases presented in the previous sections allowed us to garner four significant results relating to the research questions.

### 5.1 Result 1: in DS, the key role for policy-making is that of the translator; in simpler cases, this role can be assumed by the project manager; when the policy need is more vague, an additional role is also needed

The three cases made it clear that the most important role in the process of generating value from data is what is known as the *translator*. In the cases illustrated, this role was taken by the project manager, who needed to dedicate a lot of time in translating the work of the data scientists for the policy-makers and vice-versa. In addition, the translator had to cope with the additional problem of less interaction with policy-makers than is normally the case. In all three cases, the translator was only able to have two to four meetings with the policy-makers throughout the project.

At the start, the translator needs to understand the problem, the target population, the political risk, the financial implications and the legislative ramifications. These policy needs and constraints must then be translated into a set of questions and objectives for the DS team. In this study, the three projects diverged, posing different challenges to the policy-makers. In E-mobility, the problem was clear and the work to redefine the objective was limited to establishing the level of analysis (at macro or micro level). In Casa Italia, the problem was also clear (the prevention of natural hazards), but the scope was very broad and the policy-maker left it up to the project manager to screen the various possibilities of intervention. At the start of the project, the problem was to group together the various types of natural hazards (earthquakes, floods, etc.) and other variables were then progressively introduced, including the level of analysis (by building, municipality, province or region). The translator needs to connect the different parties and run several simulations in order to transform the translation into possible policy-based interventions. Key here was the translator's good grasp of legislative boundaries and background in accounting and finances, which was highly beneficial in simulating the project and establishing its feasibility. This required going back to the data scientists and asking them to revise their work, as well as being prepared to explain the whole thing in the “15-minute time slot” granted by the Prime Minister.

Urbanscope was the most critical case, and the project manager struggled in his role as translator. The vagueness of the research problem and the complexity of the data together led to dozens of possible settings being put in place just for the direction of the policy. The lack of any continuous interaction with the policy-maker was soon seen as an issue and the project manager wanted to leave several paths open, but this was not sustainable within the DS cycle. He then had to find a compromise between the data science potential and the policy direction to be taken. At this stage, the need for more continuous interaction and discussion with the data scientists - to enter into the detail of many issues - was not compatible with the project manager's commitments or his primary task of delivering a high-quality output to the policy-makers. This situation resulted in the management researcher being enlisted on the project as team facilitator.

To summarize, the cases indicated that a key role emerged, that of translator, and in the three projects this was taken by the project manager. The importance of the translator increases when the particular policy problem is more vague. Because the policy-maker granted the translator so few meetings, the latter had to check out whether the policy directions were compatible with the financial and legislative constraints and verify with the DS team whether the data could support different paths. When this situation became apparent, a new role emerged, similar to the *scrum master* role in agile methodology, that is, a facilitator for the team who manages the process and the way information is exchanged.

### 5.2 Result 2: among the DS phases, data acquisition emerged as particularly critical in policy-making, requiring a dedicated role

In all three cases, data acquisition was a crucial phase. When data are used for policy-making purposes, they need to be sufficiently precise for that end, have proper coverage with respect to the target beneficiaries and be provided on a continuous basis throughout the policy cycle. Two issues, in particular, are usually neglected within big data cycles and in previous experimentations:

• Data Access: total open access to data is taken for granted; the cases have instead shown that even for open data provided by the public sector, the level of aggregation was not sufficient for a precise public policy; the necessary data at a more elementary level was available but restricted.
• Data Providers: open data provided by private operators (e.g. Twitter, telecommunications companies) are usually “selective” and the operators are not totally transparent about how their sampling methods work.

The three cases cover several situations in terms of type of data and providers. Urbanscope was the most complex case, with use being made of both open and restricted data provided by a range of public and private operators; E-mobility was the simplest, and only dealt with open data; Casa Italia was an intermediate case, and there the data used was restricted and owned by public providers only (national agencies). The situation is summarized in Table 4.

**Table 4:** Data types and ownership.

	Urbanscope	Casa Italia	E-Mobility
*Data Owner*	Private and Public	Public	Public
*Data Access*	Open and Restricted	Open and Restricted	Open

As stated, E-mobility was the simplest case, and only involved public open data. In Casa Italia, there was instead the problem of gaining access to restricted data held by public providers. This was ultimately given because the request was supported by the Prime Minister, the policy-maker in this case. A second element was also crucial to ensuring trusted and continuous access, that of appointing a *data negotiator* who handled interactions with the data owners. The data negotiator was one of the statistical scientists, to secure a competent interface with the other agencies, and the project manager opted for a researcher with experience in complex settings who had demonstrated high negotiating capabilities.

Urbanscope was the richest case in terms of data, but also the most intricate, due to the presence of several digital data sources. The team was unable to ensure continuity and completeness in the provision of data, a fact that confirmed the findings that had been obtained for Casa Italia. Weaker support from policy-makers was certainly the main factor in the difficulty of reaching an agreement, but the lack of a person appointed specially to spend the necessary time and effort on screening the various options was also an issue.

To summarize, adopting DS for policy-making means putting substantial time and attention into acquiring data, this not being a one-off occurrence, but a continuous process. The exercise requires a dedicated role, held by someone able to:

• Map the initial data situation and its evolution in terms of access and data providers (Table 1)
• Engage with data owners and policy-makers to negotiate access to data, while ensuring privacy to providers and quality to policy-makers.

The following figure shows the increasing importance of the data negotiator within the scope of the data.

### 5.3 Result 3: data visualization is an essential value to build the translator's confidence; the value of visualization increases with the scope of the data

Since the early stages of big data [44], it has been claimed that data visualization is key to inspiring new answers and even new questions. The findings of our study show that this phase is less creative than in other types of setting and that the target users are not policy-makers. Policy-makers give their interlocutors a “15-minute time slot”, and in this time they have to get them to understand whether the analysis carried out by the data scientists adds value or not to a policy being or yet to be constructed. The policy-makers have no time to navigate through the features, even if visualizations are developed to help them. The same visualizations are instead pivotal for the person interacting with these policy-makers and they have to master the final output and all the key issues in the DS process. User-friendly visualizations of big data allow these intermediaries to explore data by themselves, prepare questions for the data scientists and dispel the obscurity veiling the data elaboration process and, on a positive note, build up their own confidence. More specifically, the empirical evidence of the three cases revealed two elements around which such confidence is built:

• The hypotheses underpinning the data analysis model, which are needed to verify whether the analytical choices are coherent with policy-related indications and priorities. A visual output forces both intermediary and analysts to translate their hypotheses into a common and easily intelligible language.
• The data utilized, which must cover the target beneficiaries, especially in terms of territory. In this study, all three projects used maps to show the value added by geo-referenced visualizations.

The disparity between the three cases highlighted the importance of using visualizations in different situations, where the greatest gain is when data is highly heterogeneous. Urbanscope presented the most complex situation, and it required some effort to come up with the right visualizations, as well as the navigation functions needed by the project manager to explore and analyse data. Casa Italia embodied an intermediate case, where a heat map was developed to display a given situation, but here a simple function was sufficient to explore the data. Lastly, E-mobility expressed the most straightforward case, where a simple map was all that was needed to satisfy the project manager's requirements in terms of exploring data.

### 5.4 Result 4: the outcome of the process is influenced by the “traditional” quality of data, the clarity of policy options and the way data contributes towards simulating financial and legislative effects

E-mobility and Casa Italia concluded with a legislative act that put the DS output into action. Urbanscope, the most sophisticated in terms of the DS process and output, has not yet reached this point. The difference between the three cases meant that we were able to extrapolate more general considerations and, more specifically, ideas about the influencing factors. The first element influencing the policy-maker's decision to leap into action was the “traditional” quality of the data. Urbanscope relied massively on digital data (web sources, mobile phone data) which are of lower quality if evaluated in traditional terms. For example, social media data have a problem of overall population representativeness at two levels. First, people active on social media do not reflect the entire population and there are no official statistics that can be used to infer data coverage. Furthermore, social media providers are not totally transparent about their policy for accessing public data, adding a second layer of uncertainty. This situation can be accepted when data are used for internal use or public dissemination purposes [30], but it becomes critical when data must be used for public interventions with financial and/or legislative consequences.

A second factor influencing the use of data is the clarity of the options brought to the policy-makers. DS uses data to construct sounder policy options where the various scenarios play an important role. DS becomes even more critical in complex and contentious settings. All three cases include examples of this complex decision making and the options generated through DS; however the final scenarios submitted to the policy-makers in the three cases introduced different degrees of openness. Urbanscope, with its interactive advanced platform, provided decision-makers with the means to explore many settings by applying different filters and features. In the other two cases, the policy-makers were presented with clearer options and situations. This apparently less stimulating second choice was more black-and white as well as being more compatible with the “15-minute time slot” accorded by the policy-makers.

The third factor that influence the outcome is therefore the link with the potential interventions. More precise outcome is needed to spur policy-makers into action, giving them the means to assess the implications of any proposed intervention clearly and comprehensively. Casa Italia and E-mobility provide good examples in this respect. In both cases, the outcome did not consist of the resulting data, but rather it suggested interventions underpinned by the simulation of financial needs, its compatibility with legislation and the coverage of the population within the territory in question.

## 6 Conclusions

This paper studied three real cases, investigating how big data and DS can be applied to policy-making. The claim made in several studies [45] is that there is an urgent need for this kind of application, but there are very few studies that examine the technical aspects of big data alongside the practical difficulties of introducing big data into policy cycles, and even fewer that study these matters through the simultaneous application of qualitative and quantitative analyses.

The cases, conducted through action research methodology, removed the veil from the obscure procedure of matching the demand and the offer of data within the policy definition process. We specifically followed the framing and filtering processes utilized by data scientists, and built on previous knowledge about the importance of interaction, introducing detailed qualitative observations and determining the type of roles needed and how these roles contributed to the DS phases. Three types of roles emerged from the study. Of these, the first, *translator*, plays the most crucial role. Translators are responsible for maintaining continuous alignment between the policy needs and the work of the data scientists, and they have to translate the results of this work into a form intelligible to the policy-maker within a limited time frame while making sure that all financial, legislative and policy impacts are clear. When the data scope increases, a further role is needed, that of the *data negotiator*, the second role discussed. It became clear that a solid statistical background is important in this role, as well the set of soft skills necessary to negotiate with the right actors. The third role perceived as valuable with increased ambiguity or vagueness is referred to here as the *scrum master*, drawing from agile methodology. The scrum master is responsible for facilitating interaction and exchange of information within the DS team.

These roles are then linked to the phases, which have been usually treated as equally important. Our research, however, came to the conclusion that one phase outranks the others in terms of importance for determining policies, that of data acquisition. This phase is fundamental for ensuring confidence in the policy, by providing solid data on a continuous basis. A second phase was shown to be less of a priority for policy-makers but more of a support to the translator, that of visualization. With complex data, a more sophisticated visualization tool means being able to capture new insights and opportunities. On the other hand, policy-makers have not the time for surfing data and all they want is a short report to use as the basis for furthering action.

These findings open new avenues for research, starting from the acknowledgement that the policy process and outcome vary according to the data employed and the level of vagueness or ambiguity surrounding the policy need. The results showed that there is the need to think and plan carefully for big data cycles, simultaneously combining technical aspects with organizational considerations. Data scientists take decisions in some of the phases in the big data cycle that are not neutral in terms of their results [45, 46], starting from the selection of data. Data scientists and policy-makers must establish a good dialogue, as only the latter can truly understand the implications of the policies settled upon and the outcome of these policies, or even, and sometimes more critically, those of the negotiations that must be pursued to achieve these policies.

The three roles presented, especially that of the translators and data negotiators, emerged as being key in DS applied to public policies. It is crucial, however, to point out that they are not purely technical functions: their organizational and relational capabilities are as important as their technical competence. They are the interpreters of the policy-makers’ needs, are involved in the data negotiation processes and use data to build higher “precision” into the definition of options and scenarios. This precision-focused policy is a great advancement in the public sector, but requires introducing new human resources capable of creating trust and dialogue, alongside DS technicalities.

More cases must be studied to confirm the explorative results set out in this paper, pursuing a quantitative or qualitative method. Furthermore, the actual makeup of these three roles suggests that there is the need for deeper investigation into the skills and expertise required as well as examining whether the government is fully prepared to embrace these roles. Finally, it is important to highlight the limits of these study, which can open further directions of investigation. This was an explorative study, hence the types of roles involved and the importance of the various process phases were aspects discovered late on in the process. Because of this, the authors did not work specifically upon this matter within the action research cycle, and an active and targeted interventionist approach could have modified the outcome. The findings of our study open the possibility to build real experiment, testing the performances of roles defined in different situations.

## Declarations

### Author contribution statement

Michela Arnaboldi, Giovanni Azzone: Conceived and designed the experiments; Performed the experiments; Analyzed and interpreted the data; Contributed reagents, materials, analysis tools or data; Wrote the paper.

### Funding statement

This research did not receive any specific grant from funding agencies in the public, commercial, or not-for-profit sectors.

### Competing interest statement

The authors declare no conflict of interest.

### Additional information

No additional information is available for this paper.

## Footnotes

1 We refer to the original three “V”s which are the most strictly related to the data scope.

2 When using the term data scientist, we refer to all the specialists involved in the DS cycle within the various disciplines of mathematics, statistics, science computing, web and visual design, and in the applications domain (public management and policy and/or specific services).

3 Our institution did not require us to have received approval for this study.

4 In the following, for simplicity, we have used the term “association” to refer to the association's intermediary.
